# CTMP, a predictive biomarker for trastuzumab resistance in HER2-enriched breast cancer patient

**DOI:** 10.18632/oncotarget.10719

**Published:** 2016-07-20

**Authors:** Yu-Chia Chen, Hao-Yi Li, Jui-Lin Liang, Luo-Ping Ger, Hong-Tai Chang, Michael Hsiao, Marcus J. Calkins, Hui-Chuan Cheng, Jiin-Haur Chuang, Pei-Jung Lu

**Affiliations:** ^1^ Graduate Institute of Clinical Medical Sciences, Medical College, Chang-Gung University, Tao-Yuan, Taiwan; ^2^ Division of General Surgery, Department of Surgery, Kaohsiung Veterans General Hospital, Kaohsiung, Taiwan; ^3^ Institute of Clinical Medicine, Medical College, National Cheng Kung University, Tainan, Taiwan; ^4^ Department of General Surgery, Chi-Mei Medical Center, Tainan, Taiwan; ^5^ Department of Medical Education and Research, Kaohsiung Veterans General Hospital, Kaohsiung, Taiwan; ^6^ Genomics Research Center, Academia Sinica, Taipei, Taiwan; ^7^ The Division of Pediatric Surgery, Kaohsiung Chang Gung Memorial Hospital, Kaohsiung, Taiwan

**Keywords:** CTMP, HER2, herceptin, trastuzumab, breast cancer

## Abstract

Trastuzumab is regarded as the primary therapy for patients with HER2-enriched breast cancer, but the pathological complete response for advanced cases is less than 30%. The underlying mechanism of trastuzumab resistance remains unclear and there are currently no conclusive biomarkers for patient response to trastuzumab. Identifying predictive biomarkers for trastuzumab response may allow treatments to be individually tailored and optimized multi-target therapies may be developed. CTMP activates AKT signaling in breast cancer and over-activation of AKT has been reported to contribute to trastuzumab resistance. In this study, we examined samples from 369 patients to investigate the correlation between CTMP expression level and patient outcome. Elevated CTMP expression was correlated with adverse outcomes in HER2-enriched patients including overall and disease-free survival as well as trastuzumab resistance. Ectopic expression of varying levels of CTMP in SkBR3 cells dose-dependently attenuated trastuzumab-mediated growth inhibition through AKT activation. In addition, inhibition of AKT signaling by AKT inhibitor IV and Rapamycin reversed CTMP-mediated trastuzumab resistance. In clinical samples, the high expression of CTMP was showed in trastuzumab non-responders and positively correlated with AKT activity. Taken together, we demonstrated that CTMP promotes AKT activation resulting in trastuzumab resistance in patients with HER2-enriched breast cancer. High CTMP expression not only predicted poor prognosis, but may also predict resistance to trastuzumab in HER2-enriched patients. Therefore, CTMP expression may be considered as a prognostic biomarker in HER2-enriched breast cancer and high expression may indicate a utility for AKT-inhibition in these patients.

## INTRODUCTION

Breast cancer is the most frequently diagnosed cancer in females [1]. It is a heterogeneous disease that contains tumor subgroups with substantial differences in biology and clinical outcome. Classification of breast cancer is based on three critical receptors, which are the estrogen receptor (ER), progesterone receptor (PR) and human epidermal growth factor receptor 2 (HER2). Diversity in the clinical outcome and response to treatments is well known [2]. HER2 is overexpressed in 15-20% of human breast cancers and this overexpression is accompanied by poor prognosis and poor response to chemotherapy [3].

Currently, trastuzumab (Herceptin^®^), a humanized anti-HER2 monoclonal antibody, is regarded as the standard therapy for in HER2-enriched breast cancer patients [4, 5]. Trastuzumab binds to the juxtamembrane region of HER2 and represses cell growth in HER2-overexpressing breast cancer cell lines [6]. However, for metastatic breast cancer patients with HER2-overexpression, trastuzumab monotherapy can only generate a 23-26% pathological complete response (pCR) [7, 8]. Adding chemotherapy to trastuzumab has produced better results [9]. For example, combining pertuzumab, trastuzumab and chemotherapy significantly improved progression free survival (12.4 months to 18.7 months) [10], and the antibody-drug conjugate, trastuzumab-emtansine (T-DM1) also improved response rates to 43.6% [11]. However, even with combination treatments, HER2-targeted therapy fails in 50-70% of patients with HER2-enriched breast cancer. The reason for this widespread resistance is not completely understood. Therefore, identifying biomarkers for transtuzamab response and understanding the underlying mechanism of trastuzumab resistance may helpful to overcome trastuzumab resistance.

HER2 is activated by formation of homodimers or heterodimers with other EGFR proteins and activates several downstream signaling pathways such as PI3K/AKT and RAS/MAPK [12, 13]. Moreover, the HER2-HER3 heterodimer is the most potent activator of downstream signaling, especially PI3K/AKT, which regulates cell growth and survival [14]. Trastuzumab binds to Her2 and leads to AKT repression and apoptosis in breast cancer cells [12]. However, a critical issue in trastuzumab treatment is that resistance can be conferred by over-activation of downstream AKT signaling by other mechanisms [15].

Carboxyl-terminal modulator protein (CTMP) has been reported to activate multiple signaling pathways which impinge on AKT signaling to serve roles as both as a tumor suppressor or an oncogene [16–19]. In addition to data from our lab, Ono and colleagues showed that CTMP activates AKT signaling and contributes to oncogenesis through a direct interaction in breast cancer [18, 19]. Accordingly, we hypothesize that AKT signaling activated by CTMP contributes to trastuzumab resistance in breast cancer.

In the current study, we evaluated samples from 369 patients and performed cohort analysis to reveal that high CTMP expression is associated with poor overall survival (OS) and disease-free survival (DFS). After subdividing patients into Luminal A/B, HER2-enriched and triple negative breast cancer (TNBC) subgroups based on ER, PR and HER2 status, we found that this correlation was especially strong in HER2-enriched patients compared with other subtypes. Moreover, we report that overexpression of CTMP increases trastuzumab resistance and silencing promotes trastuzumab sensitivity by modulating AKT signaling. Overall, we have uncovered a novel mechanism for CTMP-mediated trastuzumab resistance via AKT signaling. Our results suggest that CTMP may be useful as a biomarker to predict trastuzumab response and knowledge of the mechanism for this resistance can be used to develop new treatment strategies for HER2-enriched patients.

## RESULTS

### High CTMP expression is associated with poor prognosis and early recurrence in breast cancer patients

**Figure 1:**
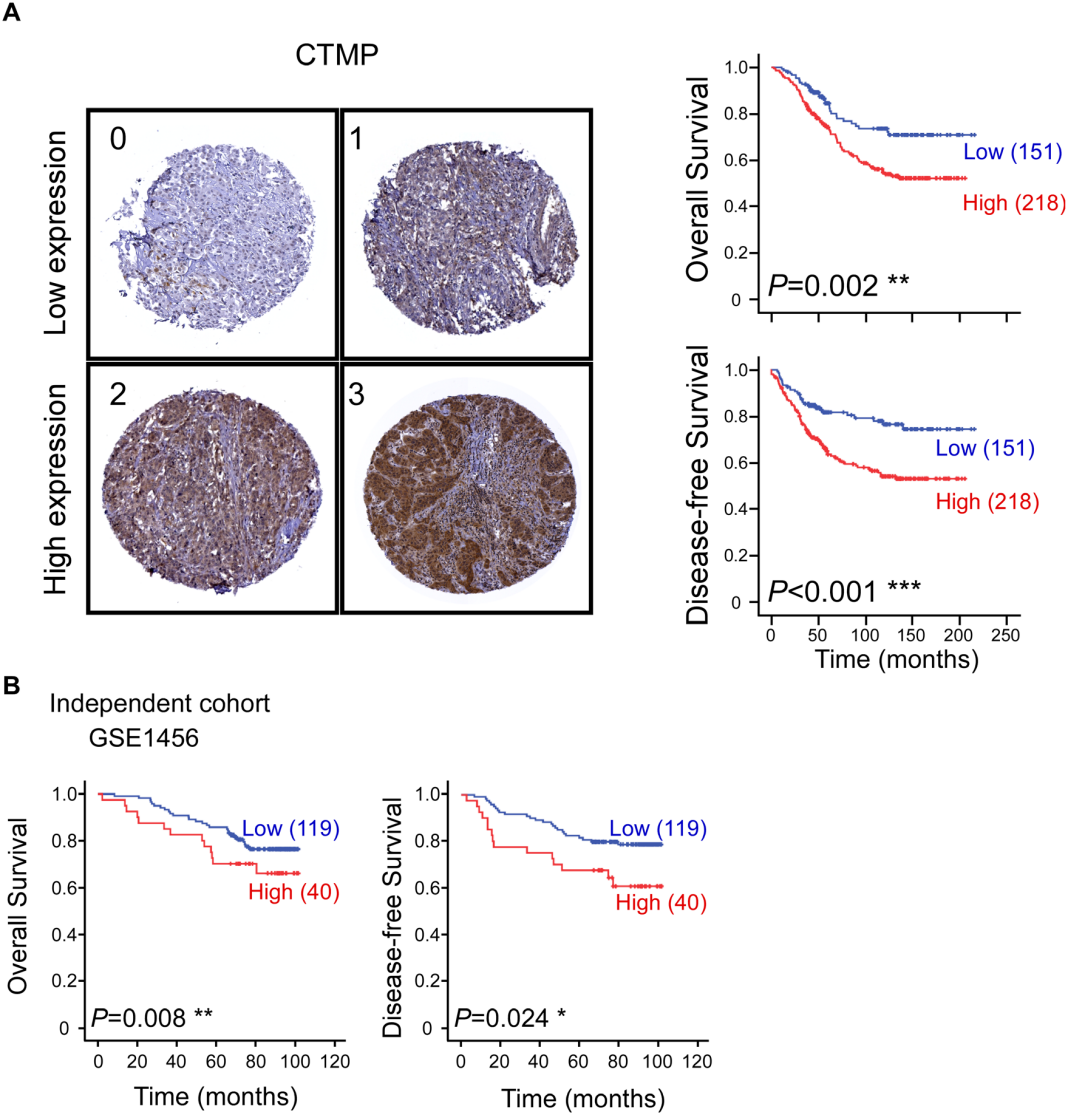
High CTMP expression is associated with poor survival and early recurrence in breast cancer patients **A**. Representative IHC images of breast cancer specimens showing CTMP expression scores from 0 to 3. Overall survival (upper) and disease-free survival (lower) of 369 breast cancer patients were stratified with CTMP expression level by Kaplan-Meier analysis. **B**. Overall and disease-free survival was confirmed in an independent cohort obtained from the GEO database (GSE1456).

The 369 patient tissue microarrays (TMAs) were used to analyze the association between CTMP expression and clinical outcome. The cohort was further subdivided by expression of three receptors, ER, PR and HER2. High expression of ER and PR were associated with better survival and high expression of HER2 was associated with poor survival (Figure S1), consistent with previous studies. To further analyze the effect of CTMP protein expression, IHC staining of CTMP was scored on a scale from 0 to 3 and subdivided into low (0 and 1) and high (2 and 3) expressing groups. Based on IHC scoring, CTMP expression and clinical information was examined by cross table (Table 1). Notably, the expression level of CTMP is significantly correlated with survival and tumor recurrence. Patients with young age and later stages tend to have high expression of CTMP, but this difference did not reach statistical significance. Next, the associations between CTMP expression and overall survival (OS) or disease-free survival (DFS) were evaluated by Kaplan-Meier and Cox proportional hazard regression analyses (Figure 1A and Table 2). High CTMP expression was related to poor OS and increased risk of recurrence. To validate the prognostic role of CTMP, an independent cohort of 159 patients was obtained from a GEO dataset (GSE1456). The clinical features of validating cohort were shown in supplementary Table 1. Patients in the validating cohort were divided into two groups consisting of the upper quartile and lower three quartiles of CTMP expression. Consistent with our previous result, high CTMP mRNA is associated with poor OS and DFS in breast cancer patients (Figure 1B). Taken together, our data indicate that CTMP may contribute to adverse outcomes related to overall survival and recurrence in breast cancer.

**Table 1:** Clinical feature of tumors, subtypes and association with CTMP expression level (N=369)

Feature	N (%)	CTMP		
		Low (%)	High (%)	*P*
**Age**				0.329
<45	108 (29.3%)	40 (10.8%)	68 (18.4%)	
>45	261 (70.7%)	111 (30.1%)	150 (40.7%)	
**Stage**				0.164
I-II	259 (70.2%)	112 (30.4%)	147 (35.2%)	
III-IV	110 (29.8%)	39 (10.6%)	71 (19.2%)	
**Survival**				<0.001***
Yes	248 (67.2%)	118 (32.0%)	130 (35.2%)	
No	121 (32.8%)	33 (8.9%)	88 (23.9%)	
**Recurrence**				<0.001***
Yes	119 (32.2%)	31 (8.4%)	88 (23.9%)	
No	250 (67.8%)	120 (32.5%)	130 (35.2%)	
**Subtypes**				0.108
Luminal A	187 (50.6%)	71 (19.2%)	116 (31.4%)	
Luminal B	52 (14.1%)	18 (4.9%)	34 (9.2%)	
Her2+	67 (18.2%)	36 (9.8%)	31 (8.4%)	
TNBC	63 (17.1%)	26 (7.0%)	37 (10%)	

**Table 2:** Univariate and multivariate Cox regression analysis for patients (n=369)

Variables	OS				DFS			
	Univariate		Multivariate		Univariate		Multivariate	
	HR (95% CI)	P	HR (95% CI)	P	HR (95% CI)	P	HR (95% CI)	P
Tumor stage	3.1 (2.2-4.5)	**<0.001**	3.0 (2.1-4.2)	**<0.001**	3.7 (2.6-5.3)	**<0.001**	3.6 (2.5-5.2)	**<0.001**
ER	0.5 (0.4-0.8)	**0.001**	0.6 (0.3-1.2)	0.159	0.7 (0.5-1.0)	**0.028**	0.4 (0.2-1.0)	**0.041**
PR	0.7 (0.5-1.0)	**0.030**	1.2 (0.7-2.0)	0.486	0.8 (0.6-1.1)	0.221	1.4 (0.8-2.5)	0.291
Her2	1.7 (1.1-2.4)	**0.008**	1.7 (0.9-2.9)	0.077	1.3 (0.9-1.9)	0.157	1.1 (0.6-1.9)	0.841
TNBC	1.5 (1.0-2.3)	0.069	1.4 (0.7-3.0)	0.384	1.2 (0.8-1.9)	0.418	0.8 (0.3-1.8)	0.557
CTMP	1.9 (1.3-2.8)	**0.002**	2.1 (1.4-3.1)	**<0.001**	2.1 (1.4-3.2)	**<0.001**	2.2 (1.5-3.4)	**<0.001**

### CTMP is associated with poor survival and early recurrence especially in HER2-enriched subtype

**Figure 2:**
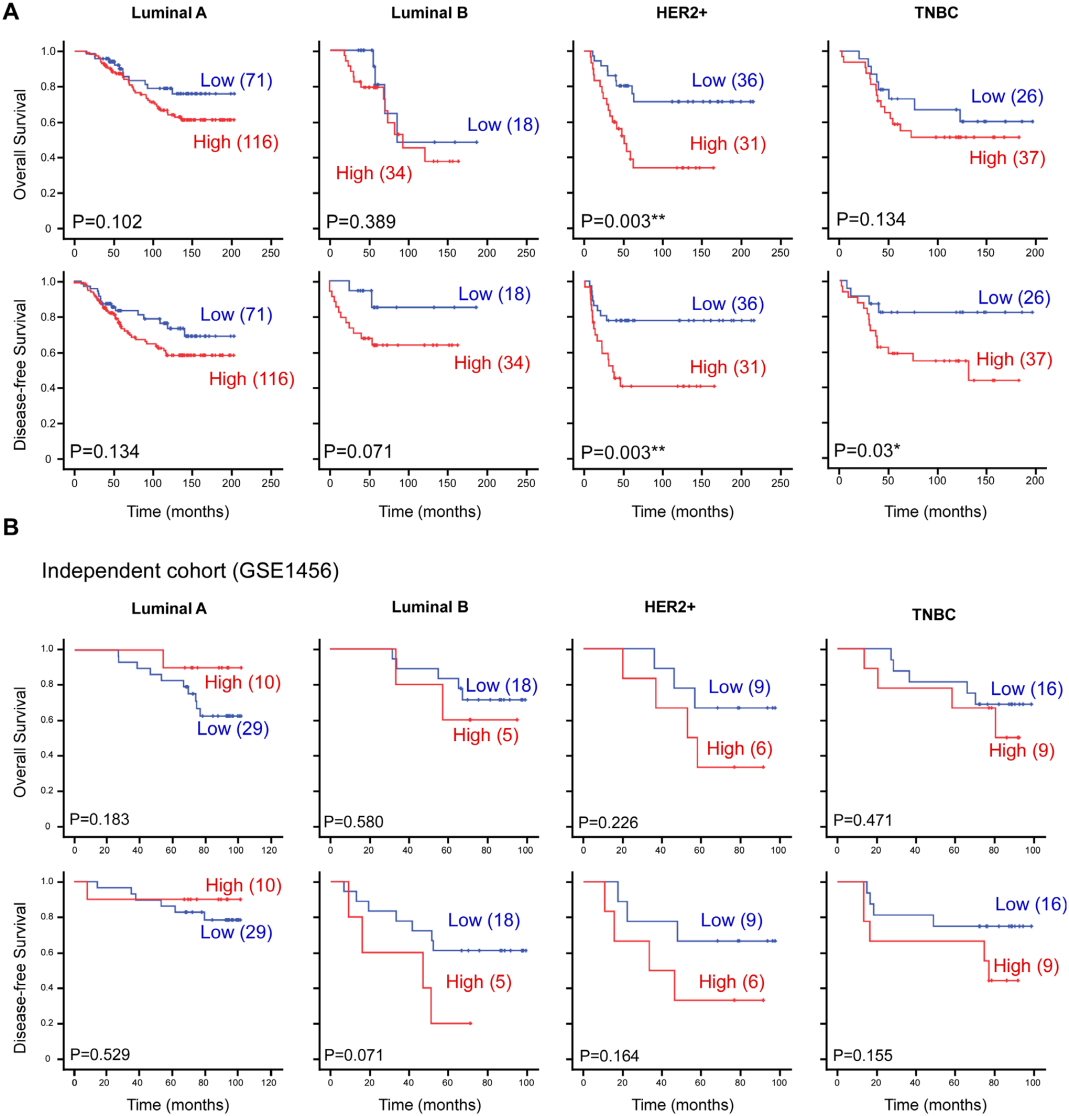
CTMP is associated with poor survival and early recurrence in HER2-enriched breast cancer **A**. The 369 breast cancer patients and **B**. 102 breast cancer patients from an independent cohort (GSE1456) were subdivided into Luminal, HER2-enriched and TNBC subgroups based on ER, PR and HER2/neu receptors. Kaplan-Meier analysis of overall survival and disease-free survival, stratified by subgroups. TNBC, triple negative breast cancer; Her2, epidermal growth receptor family member 2.

To further identify the role of CTMP in different breast cancer subgroups, including Luminal A (ER+/HER2-), Luminal B (ER+/HER2+), HER2-enriched (HER2+/ER-) and TNBC (ER-/Her2-/PR-), the association between CTMP expression level and OS or DFS were analyzed by Kaplan-Meier analysis (Figure 2A). High expression of CTMP was associated with early recurrence in all subtypes breast cancer patients. However, CTMP only significantly contributes to worse overall survival in HER2-enriched patients. Similar trends could be noted in the validating cohort but did not achieve statistical significance (Figure 2B). Trastuzumab is the primary therapy for HER2-enriched breast cancer patients. To investigate the role of CTMP in trastuzumab response, CTMP expression level was analyzed in a cohort with 48 patients (GSE76360), which contained 6 non-responders, 33 partial responders and 9 complete responders. High CTMP expression corresponded to a trend toward trastuzumab resistance (Figure S2). These findings imply that resistance to trastuzumab may be at least partially mediated by CTMP.

### CTMP opposes trastuzumab through activation of AKT signaling in HER2-enriched cells

**Figure 3:**
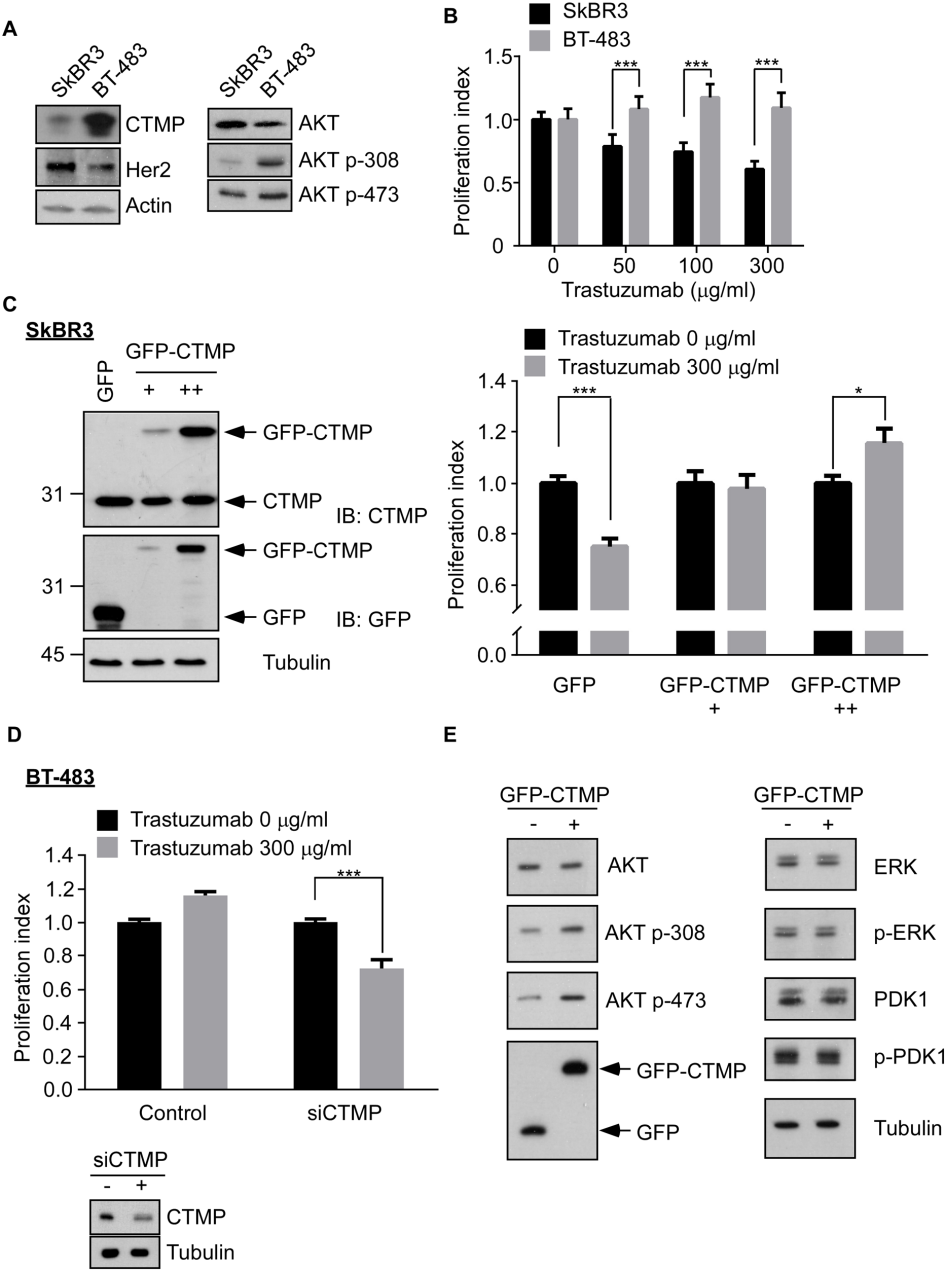
CTMP contributes to trastuzumab resistance **A**. CTMP, HER2 and AKT activity were analyzed in SkBR3 and BT-483 by Western blot. **B**. After 5 days trastuzumab treatment to SkBR3 and BT-483 cells, proliferation was examined using the MTT assay. Ectopic overexpression of different levels of CTMP in SkBR3 cells **C**. or knockdown of CTMP in BT-483 cells **D**. was used to modulate trastuzumab response as measured by the MTT assay. **E**. Western blots were performed to evaluate AKT, ERK and PDK1 and their activation in SkBR3 cells after transfection with GFP or GFP-CTMP. mean ± SEM; *, P < 0.05; ***, P < 0.001; n.s., no significance.

Previous studies have shown that activation of signaling pathways downstream of HER2, including PI3K/AKT and RAS/MAPK, may participate in conferring trastuzumab resistance [12, 15, 20]. Because CTMP was reported to promote AKT activation in breast cancer [18, 19], we hypothesized that CTMP may contribute to trastuzumab resistance through activation of AKT signaling. CTMP, HER2 and AKT status were examined in HER2 positivebreast cancer cell lines (SkBR3 and BT-483) using Western blotting (Figure 3A). Moreover, trastuzumab response was examined in BT-483 and SkBR3 by MTT assay. SkBR3 (CTMP low expression) cells were more sensitive to trastuzumab than BT-483 (CTMP high expression) (Figure 3B). Next, CTMP was overexpressed to different levels in SkBR3 cells followed by 5-day trastuzumab, and the MTT assay was used to determine cell proliferation. Ectopic expression of CTMP dose-dependently overcame trastuzumab-mediated growth inhibition in SkBR3 cells (Figure 3C). In contrast, knockdown of CTMP increased trastuzumab-mediated growth inhibition in BT-483 cells (Figure 3D). In addition, ERBB2 was overexpressed with or without CTMP overexpression in MB-231 cells (a triple negative breast cancer cell line). Again, CTMP restored trastuzumab-mediated growth repression in HER2-overexpressing MB-231 cells (Figure S3). In order to investigate the cell signaling related to CTMP-mediated trastuzumab resistance, the activation status of PI3K/AKT and RAS/MAPK pathways were analyzed in CTMP-overexpressing SkBR3 cells. Ectopic overexpression of CTMP enhanced AKT phosphorylation on T308 and S473 but not total AKT, indicating that CTMP promotes AKT activation. However, the activity of ERK and PDK1 were not changed in CTMP-overexpressing SkBR3 cells (Figure 3E). Taken together, these results suggest that CTMP-mediated tratuzumab resistance may occur through promotion of AKT activation.

**Figure 4:**
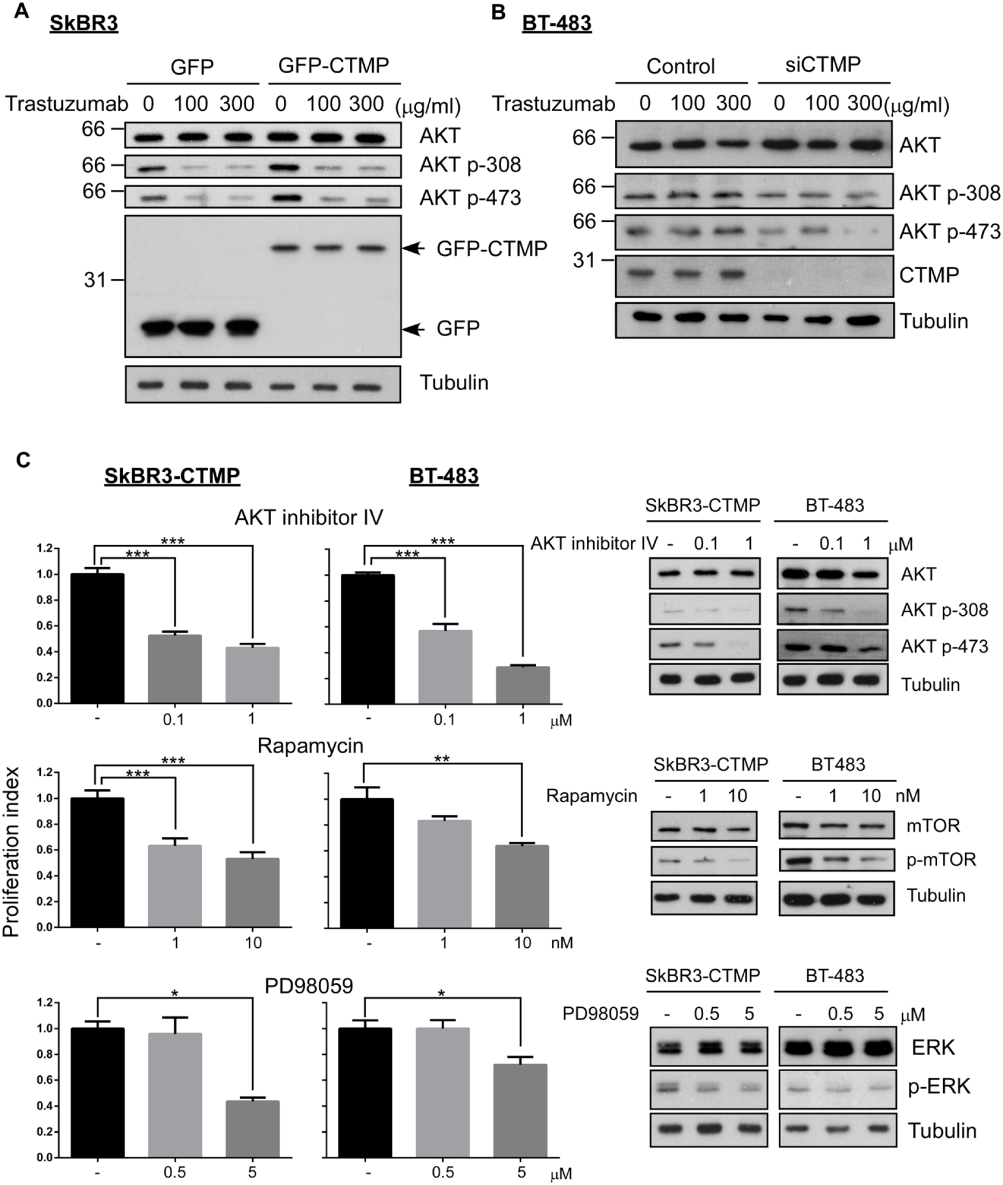
CTMP promotes trastuzumab resistance by activating AKT signaling Western-blot was performed to determine AKT activity in CTMP-overexpressing SkBR3 **(A)** and in CTMP-silenced BT-483 cells **(B)** after trastuzumab treatment for 24 hours. **C**. CTMP-overexpressing SkBR3 and BT483 were treated with trastuzumab with or without AKT inhibitor IV, rapamycin and PD98058 for 72 hours and cell proliferation ability was determined by the MTT assay.

In order to further examine whether CTMP-mediated tratuzumab resistance involves AKT, the AKT activation status was examined in CTMP-manipulated SkBR3 or BT-483 cells with or without trastuzumab treatment. AKT phosphorylation at T308 and S473 were inhibited by trastuzumab treatment, but this inhibition did not occur when CTMP was ectopically expressed in SkBR3 cells. (Figure 4A). In contrast, knockdown of CTMP in BT-483 cells promoted the effect of trastuzumab on AKT inhibition (Figure 4B). To investigate whether AKT signaling is the major contributor to CTMP-mediated trastuzumab resistance, AKT inhibitor IV, PD98059 and Rapamycin (AKT, MAPK and mTOR inhibitors, respectively), were applied to CTMP-overexpressing SkBr3 or high endogenous CTMP BT-483 cells and trastuzumab resistance was evaluated (Figure 4C). The data revealed that in the presence of AKT or mTOR inhibitors, CTMP-induced trastuzumab resistance did not occur. Moreover, treatment with the AKT inhibitor enhanced trastuzumab sensitivity even at low doses in both SkBR3-CTMP and BT-483 cells. In contrast, combination treatment of trastuzumab with MAPK inhibitor could decrease CTMP-induced trastuzumab resistance only in high dose treatment. Taken together, these data demonstrate that CTMP-mediated trastuzumab resistance is majorly mediated through activating AKT signaling.

### Patients with higher CTMP expression tended to be refractory to trastuzumab treatment

**Figure 5:**
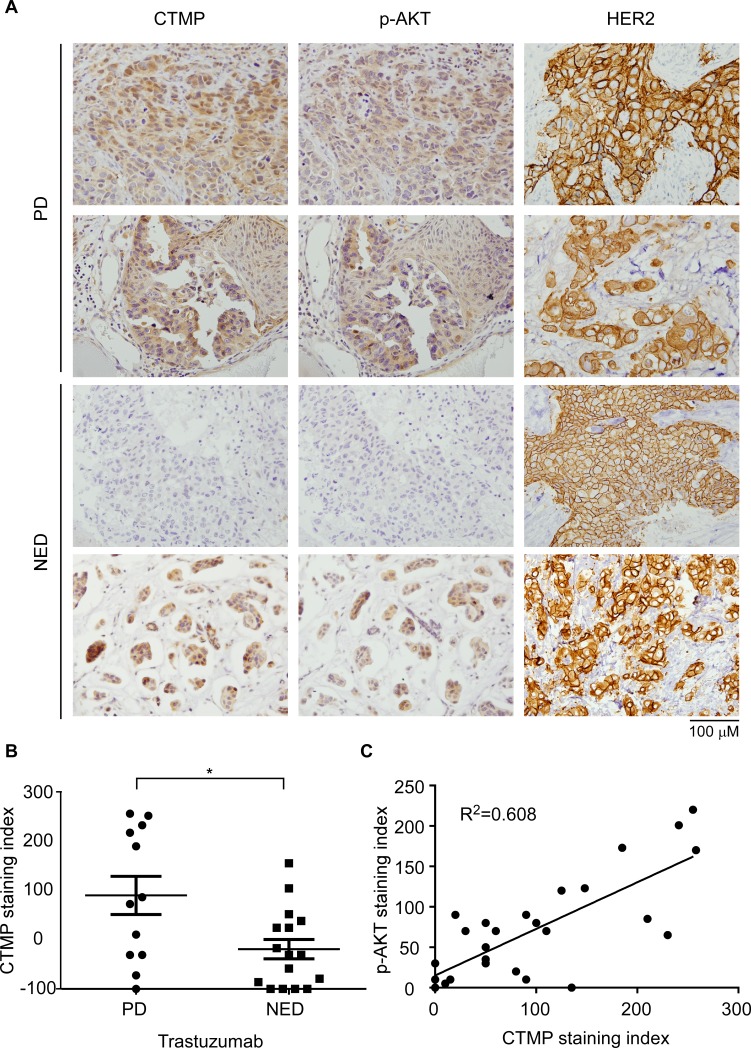
CTMP expression correlates with trastuzumab resistance in clinical samples **A**. CTMP, p-AKT and HER2 expression level were examined in HER2-enriched patients by IHC. In each sample, 100 cells were scored from 0 to 3 and the sum was taken as the staining index. **B**. The expression levels were compared between trastuzumab responders and non-responders by student *t* test. **C**. Person's correlation was used to analyze the correlation between CTMP and p473-AKT. PD: progressing disease; NED: non-evidence of disease; mean ± SEM; *, P<0.05.

In order to test the relationship between CTMP expression and trastuzumab resistance in a clinical setting, CTMP expression was examined in samples from 26 patients who received trastuzumab treatment as adjuvant or salvage treatments. Among these, 16 patients had no evidence of disease recurrence (NED) and 12 patients were refractory to trastuzumab treatment (PD). The expression levels of CTMP, Phospho-AKT and HER2 were evaluated by IHC (Figure 5A). The data showed that CTMP expression is significantly higher in tratuzumab non-responders than in trastuzumab responders (P=0.012) (Figure 5B). Pearson's correlation analysis showed that CTMP expression was inversely correlated with trastuzumab response (r = 0.468, p=0.012). In addition, CTMP expression positively correlated with AKT activity (R^2^=0.608), suggesting that CTMP may promote AKT activation *in vivo* (Figure 5C). Taken together, the results demonstrated that high CTMP expression may contribute to trastuzumab resistance through activation of AKT in HER2-enriched breast cancer patients.

## DISCUSSION

Our previous study demonstrated that CTMP is an oncogenic driver in breast cancer via positive regulation of AKT phosphorylation [19]. In this study, we evaluated 369 patients from two medical centers to investigate the predictive value of CTMP expression on clinical outcome in breast cancer. We found that high CTMP expression correlates with reduced recurrence free survival in luminal A and B, HER2-enriched and TNBC patients. However, high CTMP expression only correlated to poor overall survival in HER2-enriched patients (Figure 2A). Because all the cohorts enrolled patients during a time period when trastuzumab was not reimbursed by the National Health Bureau, only 26 patients received trastuzumab as a therapy. Analyzing these 26 patients, we found that those with high CTMP expression tended to have been refractory to trastuzumab treatment (Figure 5). This phenomenon was especially prominent with regard to visceral metastases. Consistent with the data from clinical samples, CTMP also contributes to trastuzumab resistance *in vitro* (Figure 3).

The HER2-enriched subtype was once considered to be an intractable form of breast cancer [21, 22]. However, in the era of trastuzumab, the prognosis of HER2-enriched patients has improved tremendously [23, 24]. Despite this improvement, the pCR rate of trastuzumab monotherapy in metastatic breast cancer is only 20-30% [7, 9], and even in combination with cytotoxic drugs such as cisplatin and paclitaxel, the pCR rate is does not reach above 50% [10]. There are at least three possible mechanisms of trastuzumab resistance including (1) loss or masking of trastuzumab binding sites on HER2. Trastuzumab directly binds to extracellular domain IV of HER2. However, a common HER2 mutation can generate a truncated isoform called p95HER2, which lacks the extracellular domain and possesses constitutive kinase activity [25]. Accordingly, activity of this mutation isoform is not affected by trastuzumab treatment. In addition, Mucin-4, a highly O-glycosylated membrane protein, is a putative binding partner of HER2, and their interaction may mask the trastuzumab-binding site on HER2 to promote trastuzumab resistance [26]. Activation of STAT3 has been reported to enhance Mucin-1 and Mucin-4 expression and lead to trastuzumab resistance [27]. (2) Activation of HER2 downstream signaling pathways, such as PI3K/AKT, is known to result in trastuzumab resistance. PI3KCA activating mutation or PTEN deficiency was observed in 20-25% of HER2-enriched patients [13]. Activation of PI3KCA or loss of PTEN constitutively actives AKT signaling and results in inefficient trastuzumab treatment. (3) Failure to trigger an immune response against cancer cells has been suggested as another mechanism of trastuzumab resistance, but the definite mechanisms are not well established. In addition, an IL6 inflammatory loop has been reported to expand the cancer stem cell population in HER2-positive breast cancer and lead to trastuzumab resistance [28].

To address these resistance mechanisms in HER2-enriched patients, multiple strategies have been employed in clinic. Other HER2 antibodies, including Pertuzumab, T-DM1, and lapatinib may overcome the masking of the trastuzumab-binding site [10, 29–31]. Anti-PD1 (programmed death-1) monoclonal antibody may rescue the failure to generate immune response [32]. Phosphorylated AKT is frequently associated with poor prognosis in HER2-enriched patients [15]. Our study showed that ectopic expression of CTMP contributes to trastuzumab resistance in a HER2-enriched cell line (Figure 3C and 4A). On the contrary, silencing CTMP in a HER2-enriched cell line, BT-483 enhances trastuzumab response (Figure 3D and 4B). Thus, targeting AKT signaling may be a viable strategy for overcoming trastuzumab resistance as well.

As an important downstream effector of both HER2 and estrogen receptors, AKT plays a critical role for tamoxifen and trastuzumab resistance [33]. A suite of clinical trials has targeted both upstream and downstream components of the AKT signaling pathway and AKT itself. For example, BKM-120, an oral pan-class 1 PI3K inhibitor is known to produce an objective response and its potential as a neoadjvaunt is under examination in the neoPHOEBE trial (NCT01816594, US gov identifier). Up to now, the therapeutic effect of direct AKT inhibitors, such as perifosine [34], has been unsatisfactory. New inhibitors, including MK-2206 (Merck) [35], and GSK-2141795 (Glaxo-SmithKline) are under evaluation (ClinicalTrials.gov Identifier: NCT01902173). Everolimus is an inhibitor of mTOR, which is a downstream target of AKT. This molecule has shown promise [36], but compared to the excellent safety profile of trastuzumab, hematological complications and noninfectious pneumonitis related to everolimus use have warranted caution in clinical application.

Since CTMP contributes to trastuzumab resistance through AKT phosphorylation (Figure 4A and 4B) and this activation preexists in trastuzumab-naïve patients (Figure 5), it is important to identify the HER2-enriched patients with high CTMP expression and tailor a different therapeutic strategy for them. In this case, concomitant or sequential treatment with effective AKT singling inhibitors and trastuzumab should be considered. It is common to combine chemotherapeutic agents with a variety of biological functions, such as antimetabolites, anti-mitosis, etc. For example, LHRH analogue given concurrently with Tamoxifen does better than LHRH alone [37]. Cocktails of targeted therapeutics to counteract the diverse signaling pathways of cancer cells should be considered as well.

There is a 20-25% recurrence rate in breast cancer patients, and distant metastases occur in 60-75% of patients with recurrent cancers. For those patients with distant metastases, the 5 year survival rate is only 20% [38, 39]. Our data revealed that CTMP is highly correlated with recurrence in each breast cancer subgroup, which implies that in addition to trastuzumab response, CTMP expression may be prognostic for tumor recurrence. Based on our mechanistic data, these correlations are likely related to CTMP-mediated increases in phosphorylation of AKT, as phosphorylated AKT is strongly associated with poor breast cancer prognosis [40]. These findings also suggest that CTMP-targeted therapies may be useful in the development of new strategies to overcome trastuzumab resistant and recurrent breast cancer.

## MATERIALS AND METHODS

### Clinical specimens

The total 369 clinical specimens were collected from two different cohorts. 261 samples were obtained from Kaohsiung Veterans General Hospital (KVGH) archives with Institutional Review Board approval. The other 108 patients were collected from Taipei Municipal Wanfang Hospital (WFH) archives with Institutional Review Board approval. All HER2-enriched patients didn't receive trastuzumab treatment because no national health insurance reimbursement was provided during the cohort treatment period. In addition, samples from the 28 patients who did receive trastuzumab were evaluated to probe the relationship between CTMP level and trastuzumab response.

### Immunohistochemisty

Tissue sections (5 μm) were de-waxed and rehydrated. Antigen retrieval was performed by incubating the slides in 10 mmol/L citric buffer (PH 6.0) or Tris-EDTA (PH 9.0) and microwaving for 15 minutes. Endogenous peroxidase activity was blocked using 0.5% H_2_O_2_. After blocking, the slides were incubated with primary antibody against CTMP (Cell signaling), AKT-p473 (Cell signaling) and HER2 followed by biotin-conjugated secondary antibody, polymer-HRP and diaminobenzidine tetrahydroxychloride (DAB) solution.

### Cell culture and transfection

BT-483 and SkBR3 cell lines (ATCC) were cultured in RPMI-1640, supplemented with 10% fetal bovine serum and antibiotics. The cultures were maintained in a 5% CO_2_ humidified atmosphere at 37°C. Transfections with GFP-CTMP plasmid or CTMP siRNA were performed with Lipofectamine 2000 for 6 hours, followed by replacement of the culture medium for 24-72 hours.

### Trastuzumab treatment and MTT assay

The CTMP-manipulated SkBR3 or BT-483 cells were seeded at 5 × 10^3^ cells per well in 96-well plate. After cells attached, serum free RPMI-1640 was added for 1 hour and then replaced with 3% serum RPMI-1640 containing indicated trastuzumab concentration for 5 days. After trastuzumab treatment for 5 days, 3- (4,5-cimethylthiazol-2-yl)-2,5-diphenyl tetrazolium bromide (MTT) was diluted in culture medium to final 20% v/v and added 100 μl mixture solution in each well for 30 minutes. DMSO was used to dissolve crystals and OD570 nm was measured.

### Western blot

Lysis buffer (4°C) was added to the cells for 10 minutes, and the cell lysates were then collected, added to sample buffer and heated to 95°C for 10 minutes. The protein samples were electrophoresed by SDS PAGE to separate the proteins by molecular weight. The proteins were transferred from the gel to PVDF membranes using an immersion transfer device. After blocking with 5% milk in TBST, the membranes were incubated with the indicated primary antibodies for AKT, AKT p308, AKT p473, Actin or Tubulin overnight at 4°C. The membranes were washed with TBST 3 times for 10 minutes each time and then incubated with the appropriate secondary antibodies. X-ray films were used to detect horseradish peroxidase conjugated signaling.

### Statistical analyses

All observations were confirmed by at least three independent experiments. Data were expressed as means ± SD. The associated between overall survival (OS) and disease-free survival (DFS) were analyzed using the log-rank Kaplan-Meier and Cox regression analysis. Statistical comparisons of the results were made using student t test and Mann-Whitney test. All tests were two-sided, and p value of less than 0.05 was considered to be statistically significant. SPSS version 20 (SPSS Inc.) and GraphPad Prism 6 software were used to analyze data.

## SUPPLEMENTARY MATERIALS FIGURES AND TABLES


