# Supplementary material for: CTMP, a predictive biomarker for trastuzumab resistance in HER2-enriched breast cancer patient
Source: Oncotarget. 2016 Jul 20;8(18):29699–710. doi: 10.18632/oncotarget.10719 (PMC5444696; doi:10.18632/oncotarget.10719)
Supplement: Supplementary file 1 [file oncotarget-08-29699-s001.pdf]

CTMP, a predictive biomarker for trastuzumab resistance in HER2-enriched breast cancer patient

SUPPLEMENTARY FIGURES AND TABLE

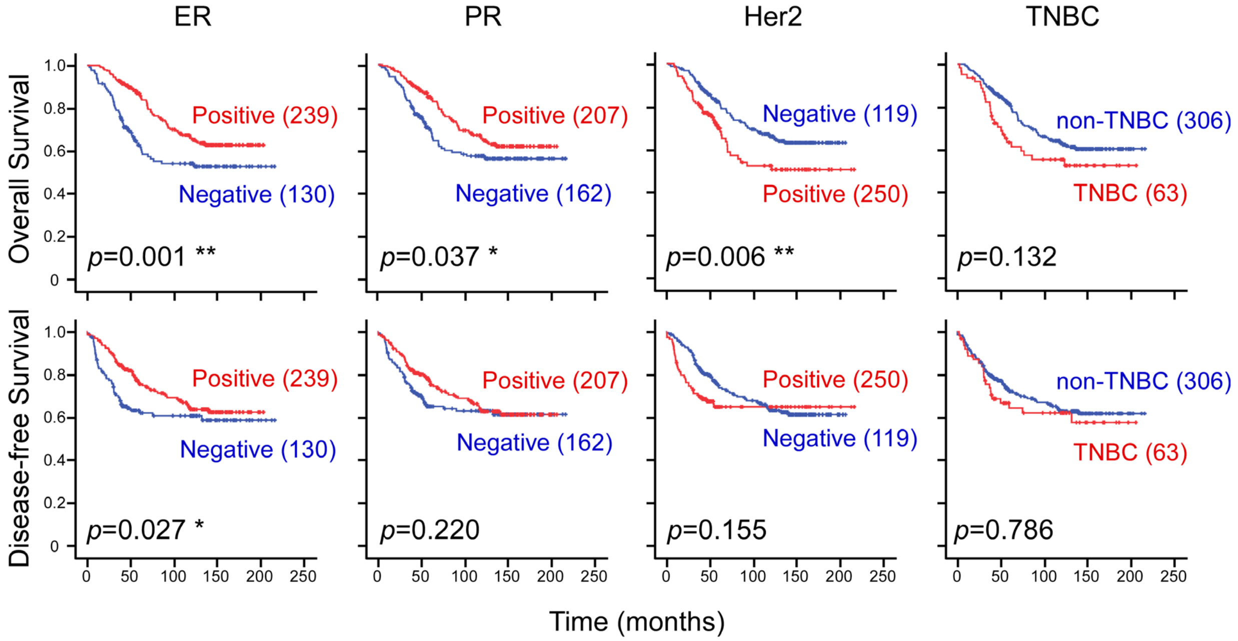

Supplementary Figure S1: Cohort examining by ER, PR, HER2 and TNBC. Overall survival (upper) and disease-free survival (lower) of 369 breast cancer patients were stratified with ER, PR, HER2 and TNBC status by Kaplan-Meier analysis.

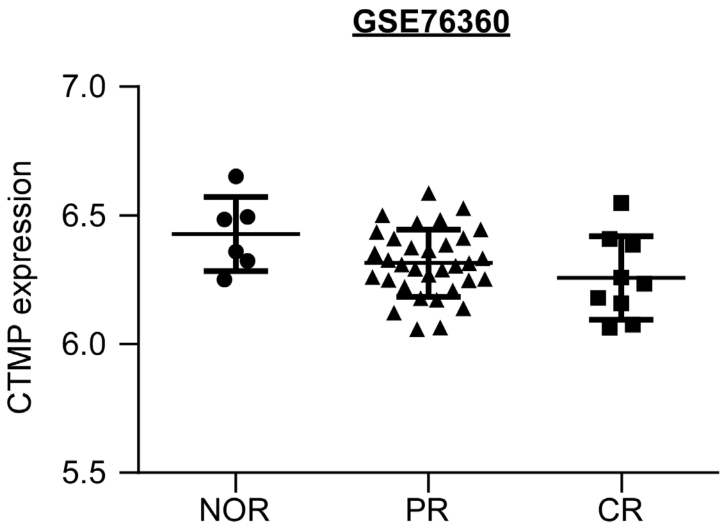

Supplementary Figure S2: High expression of CTMP is associated with trastuzumab resistance. Analysis of the CTMP expression level in different trastuzumab response groups. Samples were obtained from the GEO dataset (GSE76360). NOR: no-response; PR: partial response; CR: complete response.

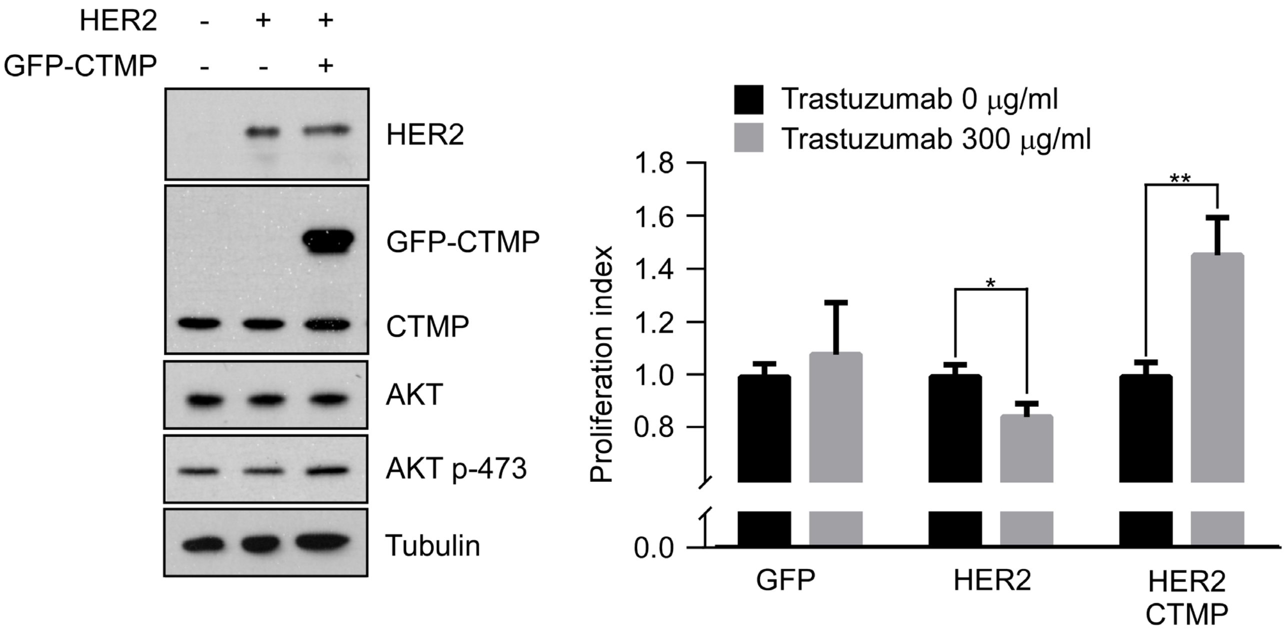

Supplementary Figure S3: CTMP overcomes trastuzumab-mediated growth inhibition in MB-231/HER2 cells. HER2 with or without CTMP was ectopically overexpressed in MB-231 cells (left), and response to trastuzumab was evaluated by the MTT assay (right).

Supplemental Table S1: Clinical feature of tumors, subtypes and association with CTMP expression level (N=159)

Feature	N (%)	CTMP		
		Low (%)	High (%)	*P*
**Grade**				0.059
1	28 (17.6%)	21 (13.2%)	7 (4.4%)	
2	58 (34.3%)	46 (28.9%)	12 (7.5%)	
3	61(36.1%)	40 (25.2%)	21 (13.2%)	
N/A	12(7.5%)	12 (7.5%)	0 (0.0%)	
**Survival**				0.216
Yes	119 (74.8%)	92 (57.9%)	27 (17.0%)	
No	40 (25.2%)	27 (17.0%)	13 (8.2%)	
**Recurrence**				**0.038***
Yes	119 (74.8%)	94 (59.1%)	25 (15.7%)	
No	40 (25.2%)	25 (15.7%)	15 (9.4%)	
**Subtypes**				0.378
Luminal A	39 (24.5%)	29 (18.2%)	10 (6.3%)	
Luminal B	23 (14.5%)	18 (11.3%)	5 (3.1%)	
Her2+	15 (9.4%)	9 (5.7%)	6 (3.8%)	
TNBC	25 (15.7%)	16 (10.1%)	9 (5.7%)	
Normal	37 (23.3%)	31 (19.5%)	6 (3.8%)	
Other	20 (12.6%)	16 (10.1%)	4 (2.5%)	
